# Q Fever Outbreak in Industrial Setting

**DOI:** 10.3201/eid1007.030536

**Published:** 2004-07

**Authors:** Hugo C. van Woerden, Brendan W. Mason, Lika K. Nehaul, Robert Smith, Roland L. Salmon, Brendan Healy, Manoj Valappil, Diana Westmoreland, Sarah de Martin, Meirion R. Evans, Graham Lloyd, Marysia Hamilton-Kirkwood, Nina S. Williams

**Affiliations:** * National Public Health Service for Wales, Cardiff, United Kingdom;; † Department of Public Health, Gwent, United Kingdom;; ‡ Department of Public Health, Cardiff, United Kingdom;; § University Hospital of Wales, Cardiff, United Kingdom;; ¶ Special Pathogens Reference Unit, Wiltshire, United Kingdom

**Keywords:** Q fever, Coxiella burnetii, disease outbreak, straw, construction materials, Wales, research

## Abstract

An outbreak of Q fever occurred in South Wales, United Kingdom, from July 15 through September 30, 2002. To investigate the outbreak a cohort and nested case-control study of persons who had worked at a cardboard manufacturing plant was conducted. The cohort included 282 employees and subcontractors, of whom 253 (90%) provided blood samples and 214 (76%) completed questionnaires. Ninety-five cases of acute Q fever were identified. The epidemic curve and other data suggested an outbreak source likely occurred August 5–9, 2002. Employees in the factory's offices were at greatest risk for infection (odds ratio 3.46; 95% confidence interval 1.38–9.06). The offices were undergoing renovation work around the time of likely exposure and contained straw board that had repeatedly been drilled. The outbreak may have been caused by aerosolization of *Coxiella burnetii* spore-like forms during drilling into contaminated straw board.

## Abstract 2

An outbreak of Q fever was likely caused by renovation work that aerosolized contaminated straw board.

Q fever is an infection caused by the bacterium *Coxiella burnetii*. The organism is found in most parts of the world and is endemic in wild and domestic animals, rodents, and arthropods, which provide a reservoir for infection (1). Most outbreaks have been associated directly or indirectly with farms or farm animals, but urban outbreaks have been described (2,3). Infected animal birth products can cause outbreaks of Q fever, and an infected placenta can contain as many as 10^9^ organisms per gram (4). *C. burnetii* produces a spore-like form, which can survive for months or years before being inhaled and causing infection (5,6). The infective dose can be as low as one organism; therefore, large outbreaks can be caused by a small source (7). A review of the literature was undertaken by one of the authors (available from H.C. van Woerden). This investigation identified 79 outbreaks reported in 48 articles in English language journals. An additional 44 papers in other languages were identified in a literature review by Williams (7) and a further 40 German outbreaks were identified in a literature review by Hellenbrand et al. (8). The literature review suggested that most outbreaks are associated with primary or secondary aerosols that arise around infected animals or contaminated fomites (5,9).

Approximately 70 cases of Q fever are identified in the United Kingdom each year as a result of routine surveillance (R. Smith, pers. comm., Zoonosis Surveillance, Communicable Disease Surveillance Centre, Wales). However, seroprevalence studies indicate that approximately 27% of farmers and 10% of the general population have antibodies, which suggests previous exposure to the organism; this finding does not appear to have changed substantially during the last 45 years (10,11). We report an investigation of an outbreak of Q fever at the premises of a manufacturer of cardboard packaging materials in Newport docks, South Wales, in the summer of 2002.

## Methods

### Description of the Outbreak

A possible outbreak of atypical pneumonia was reported to the local public health department on September 12, 2002, by a physician who reported that other employees at the patient's workplace had had similar symptoms. The outbreak was verified, an outbreak control team assembled, and a case definition agreed on (12). By September 15, 2002, a total 12 potential patients had been identified and the first case confirmed as Q fever. The investigation was begun by contacting all relevant hospital clinicians and general medical practitioners in the Gwent area and requesting that they supply blood samples from any patients who had symptoms compatible with Q fever.

### Epidemiologic Investigation

Several hypotheses were explored. An outbreak could have occurred in the wider community, and employees could have been infected by contaminated straw, hay, or compost; wild or feral animals; or domestic animals, particularly pregnant or newborn animals. Contamination could have been through sources brought into the factory, which included the following: contaminated personal belongings; contact with a contaminated source on the docks, which were on the way to work; windborne spread from infected animals on nearby farms; windborne spread from goods passing through the docks; animals or animal-based feed; contaminated hay, straw, or farm vehicles; sources in the factory premises; wooden delivery pallets contaminated with chicken carcasses returned to the factory; infection passed by red mites biting infected seagulls nesting on the roof, which then may have bitten staff in the factory; airborne spread from a cat that had given birth near the factory 2 years previously; airborne spread of contaminated dust generated by the renovation work; dust previously contaminated by an infected animal, bird, rodent, or bat; or contaminated straw or straw board aerosolized during drilling or removal.

We obtained data from a variety of sources, including a questionnaire survey, laboratories, clinicians, and factory management. A list containing details of the workforce and possible, past, and confirmed cases was developed and used to construct an epidemic curve. Data on place of work provided by factory management were used to calculate attack rates. Details were also collected on persons who had been on site for a limited number of days to help pinpoint the onset of the outbreak. Employees working on the factory floor were examined to determine whether a pattern occurred in the infected patients by calculating the relative risks for employees at each machine on the factory floor.

Two clinics were held at the factory on September 23 and 30, 2002, where blood samples were obtained from and questionnaires were completed by employees and subcontractors who had worked at the factory at any time from July 15, 2002, through September 30, 2002. Data from the questionnaires were analyzed by using a nested case-control design, where cases were defined as confirmed cases and controls were defined as noncases. The questionnaire explored risk factors in three categories: possible exposure in the community, in the docks or on the route to work, and at work.

Case definitions were applied to employees and subcontractors who had worked at the factory at any time from July 15, 2002, through September 30, 2002. A confirmed case was defined as phase 2 immunoglobulin (Ig) M > 320 or fourfold rise in complement fixation tests (CFT) titer or IgM 20–160 + phase 2 IgG > 320. A past exposure was defined as phase 2 IgG but no phase 2 IgM. A noncase (control) was defined as a CFT of < 8 + negative phase 2 IgM and IgG + either no symptoms or onset of symptoms > 7 days before blood sample or (in which the onset of symptoms was within 7 days of first sample) two consecutive blood samples with a CFT of < 8 + one negative phase 2 IgM and IgG. A possible case was defined as all remaining cases.

Data were analyzed with EpiInfo (v. 6.04, Centers for Disease Control and Prevention, Atlanta, GA), Excel 97 (Microsoft, Redmond, WA), and Stata (v. 7, Stata Corporation, College Station, TX) software. Possible cases in patients and persons with evidence of past exposure were excluded from the nested case-control study. The analysis also excluded responses of "not sure" from odds ratio (OR) calculations. We calculated Mantel-Haenszel OR with exact 95% confidence limits (CI) (13).

### Microbiologic Investigation

Complement fixation tests for phase 1 and phase 2 antibodies were performed at the Public Health Laboratory Service, Cardiff. IgM and IgG immunofluorescent assays were carried out on the samples by the Centre for Applied Microbiological Research (CAMR), Porton Down, UK. Laboratory staff monitored all requests for Q fever serologic testing from general practitioners and hospital clinicians to identify any additional cases that might be linked with the outbreak.

### Environmental Investigation

Environmental information on the factory was gathered by environmental health officers and other members of the outbreak control team during site visits on September 23, 2002, and September 30, 2002. Management representatives of several other premises in or near the docks were interviewed.

On October 11, 2002, an environmental scientist collected 17 random environmental samples of straw and dust from inside and outside the factory premises. The samples were sent for polymerase chain reaction (PCR) testing at CAMR.

## Results

### Epidemiologic Investigation

**Figure 1:**
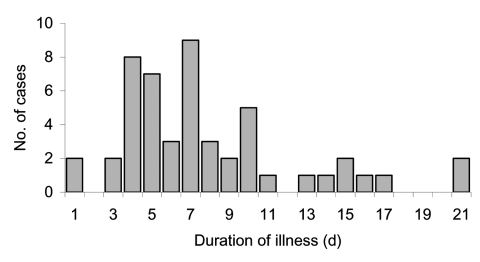
Duration of illness in symptomatic Q fever patients, Newport, Wales, August–September 2002.

A total of 222 employees and 60 subcontractors were working in the factory complex from July 15 through September 30, 2002. Questionnaires were completed by 214 (75.9%) of these 282 persons. Of the 253 persons who were tested, we identified 95 (37.5%) confirmed cases of Q fever, 42 possible cases, 8 cases of past exposure, and 108 noncases. Four persons refused blood tests but completed a questionnaire. Data for the nested case-control analysis were available on 75 (78.9%) of the 95 confirmed cases and 101 (93.5%) of the 108 noncases. The frequency and duration of symptoms are shown in Table 1 and Figure 1. Ten participants were still ill when questioned, and 5 did not provide a date of onset of symptoms. Five patients (5.3%) were admitted to the hospital with pneumonia. Some patients experienced fatigue. However, the clinical impression of one of the authors involved in follow up of patients was that very few neurologic symptoms occurred during this outbreak, compared to a previously reported U.K. outbreak (14). Further analysis of clinical symptoms is being prepared as a separate paper.

**Table 1:** Frequency of symptoms in 55 symptomatic patients with confirmed cases of Q fever, Newport, Wales, August–September 2002

Symptom	Yes (%)	Not sure
Fever	41 (75)	1
Sweats	53 (96)	0
Headache	51 (93)	1
Weight loss	26 (47)	2
Cough	24 (44)	0
Shortness of breath	25 (45)	2
Joint pain	44 (80)	3
Chest pain	20 (36)	5
Jaundice	4 (7)^a^	5

^a^These responses represent a misunderstanding of the term jaundice, since none of these persons had clinical jaundice.

**Figure 2:**
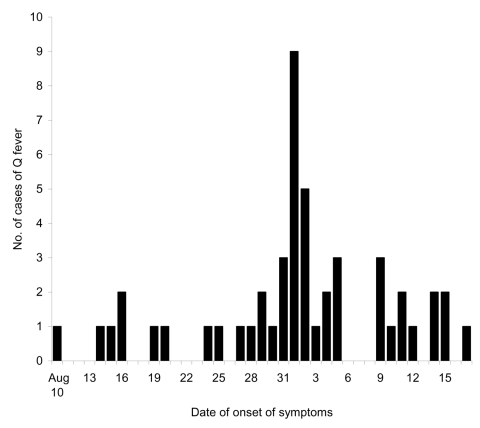
Epidemic curve for 49 confirmed cases in Q fever outbreak, Newport, Wales, August–September 2002.

The epidemic curve for 49 confirmed cases where the date of onset of symptoms was reliably known is shown in Figure 2. A peak incidence occurs around September 1, 2002. Based on an incubation period of 5 to 40 days (1,5), these data suggest that almost all the cases can be accounted for by an exposure from August 7 to 11, 2002.

Seven confirmed patients were only present in the factory on 2 or 3 days. All these persons were present in the factory and potentially exposed to infection from August 5 through August 9, 2002.

An analysis of home postal codes of 71 participants with Q fever who completed the questionnaire showed no discernible pattern and indicates that our participants were not part of a larger Q fever outbreak with a common source in the community. Details of place of work within the factory complex were available for participants with 61 confirmed cases and 81 controls. No cases occurred among persons working exclusively outside the factory floor or office block. In addition, no cases were identified among seven participants working in a separate design office, one employee working exclusively in the dispatch building, or five sales representatives who only called into the office on an occasional basis (Table 2). The OR for having a case in office staff compared with other staff was 3.46 (95% CI 1.38–9.06). ORs for groups of staff working in all other areas were <1 (Table 2).

**Table 2:** Attack rates and odds ratios (OR) for different areas of work at factory implicated in Q fever outbreak, Newport, Wales, August–September 2002

Category	No. of persons working in area			No. of persons working elsewhere			
	Cases	Controls	Attack rate (%)	Cases	Controls	Attack rate (%)	OR (95% CI)^a^

Production/factory floor	35	52	40.2	26	29	47.3	0.78 (0.36–1.57)
Dispatch	0	1	0	61	80	43.3	0 (0–71.79)
Dispatch/factory floor	4	4	50.0	57	77	42.5	0.68 (0.14–2.68)
Office	20	10	66.7	41	71	36.6	3.46 (1.38–9.06)
Production-based but sometimes in the office	1	2	33.3	60	79	43.2	0.66 (0.01–12.96)
Design	0	7	0	61	74	45.2	0 (0–0.88)
Sales representatives	0	5	0	61	76	44.5	0 (0–1.42)
Dispatch but sometimes in the office	1	0	100	60	81	42.6	Undefined

Total

61

81

43.0

^a^CI, confidence interval.

**Figure 3:**
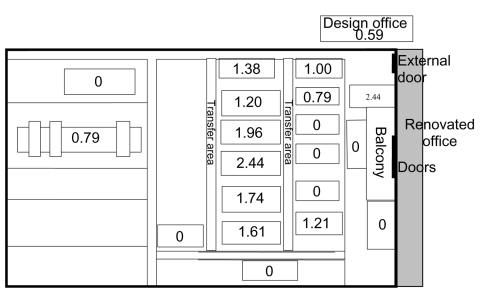
Relative risks for employees at various machines on the factory floor in Q fever outbreak, Newport, Wales, August–September 2002.

The relative risks of having a case of Q fever among the cohort of employees working at different machines on the factory floor are shown in Figure 3. The balcony in Figure 3 is not drawn to scale. It overhangs the adjacent machines where the relative risk to workers was zero. The relative risk for infection was greatest among people who worked in the center of the factory floor outside the shadow of the overhanging balcony; the risk for infection dropped towards the sides of the building.

Eighty-three percent of confirmed cases were in men, a similar male-to-female ratio to that of the cohort as a whole, and median age was 44 years (range 22–60 years). Questionnaire data indicated that infected employees did not own animals that had given birth or had a miscarriage nor had these employees had any contact with the birth products of animals. One subcontractor, who cleaned windows at the factory, also worked on a farm and had been in contact with animals that had given birth, but the evidence did not suggest that any of these had been infected with *C. burnetii*. Additionally, the serologic tests for this employee were negative for Q fever, and the dates on which he visited the factory suggest that his clothing or possessions could not have been the source of the outbreak.

Case-patients were much more likely than controls (OR 5.86; 95% CI 0.55 to 291.88) to recall coming across a hay lorry entering or leaving the docks while on their way to or from work. Adjusting for cases in those whose office was refurbished reduced the OR in those who saw a hay lorry (OR 3.00; 95% CI 0.28–31.80). Employees whose offices had been refurbished were at greatest risk for infection (OR 2.60; 95% CI 0.77–9.57). Employees who described themselves as "never near an external door or window" were more likely to be infected than those who worked "near an external door or window on most days" (OR 1.98; 95%CI 0.72–5.56). Living on a farm appeared slightly protective (OR 0.35; 95% CI 0.01–4.53) as did the regular handling of compost (OR 0.14; 95% CI 0.00–1.03). However, none of these findings, or those in Table 3, reached statistical significance value of 5%.

**Table 3:** Odds ratios (OR) for different risk factors in Q fever outbreak, Newport, Wales, August–September 2002

Exposure at work	No. of persons exposed to risk factor		No. of persons not exposed to risk factor		
	Cases	Controls	Cases	Controls	OR (95% CI)
Office refurbished	24	23	6	15	2.61 (0.77–9.57)
Never near an external door or window/near a window or door most days	13	10	40	61	1.98 (0.72–5.56)
Smoker/never smoked	15	35	42	48	0.49 (0.22–1.08)
Saw hay lorry on the docks	4	1	56	82	5.86 (0.55–291.88)
Live on a farm	1	3	72	76	0.35 (0.01–4.53)
Regularly handle compost	1	9	68	83	0.14 (0.00–1.03)
Contact with animal births or miscarriages	0	6	39	54	0.00 (0–1.26)

^a^CI, confidence interval.

The work undertaken by the seven participants with the shortest incubation times was examined for unusual characteristics. A higher proportion of those with a short incubation time were women (three of seven) when compared with the general population. Four of the seven participants worked in offices that had been refurbished, and the remaining three worked on the factory floor. Their duration of illness varied from 4 to 14 days.

### Microbiologic Investigation

Two hundred and fifty-three participants (89.7%) provided blood samples. Some participants had only one sample taken and others had up to four additional samples taken from September through December 2002 at primary care or hospital clinics. A summary of CFT and IgM results is shown in Tables 4 and 5.

**Table 4:** Summary of highest phase 2 CFT results recorded for each person in the cohort in Q fever outbreak, Newport, Wales, August–September 2002^a^

Highest CFT	AQF cases	Noncases	Past exposure	Possible cases
<8	4	104	5	35
8	4	2	3	2
16	12	1	0	2
32	14	1	0	2
64	16	0	0	1
128	21	0	0	0
256	17	0	0	0
512	5	0	0	0
1,024	2	0	0	0
Totals (253)	95	108	8	42

^a^CFT, complement fixation test; AQF, acute Q fever.

**Table 5:** Summary of highest phase 2 IgM results recorded for 107 persons in the cohort^a^

IgM P2 values	AQF cases	Past exposure	Uncertain status
0	1	8	
Low levels	0	0	1
80	0	0	2
<60	0	0	0
160	5	0	2
320	3	0	0
640	16	0	0
1,280	4	0	0
>1,280	65	0	0
Total	94	8	5

^a^Ig, immunoglobulin; AQF, acute Q fever.

As a result of informing general practitioners in the area of the outbreak, more than twice the normal numbers of general practitioner requests for Q fever serologic testing were received. Hospital samples submitted for Q fever serologic testing were also monitored. Our monitoring identified one patient with a chronic case of Q fever and one patient with an acute, neither were associated with this outbreak. No *C. burnetii* was identified by PCR testing the straw board and dust samples that were obtained from the factory.

### Environmental Investigation

The factory consists of several buildings. The main production area consists of a large, rectangular open-plan hanger with an elevated office block at one end of the rectangle (Figure 3). The office block was undergoing extensive renovation work at the time of the outbreak. This involved drilling >100 holes in the straw board ceiling to allow the attachment of a new suspended ceiling. Some internal walls made of straw board were also removed. A temporary corridor was created from plastic sheeting which ran through the area being renovated but did not form a complete seal. No respiratory protection was used by the contractors or the workforce at any stage. The corridor was in constant use by staff in adjacent offices. Office staff and factory floor workers who visited the offices consequently had some exposure to dust generated by the renovation work.

The layout of the factory is consistent with the possibility of disseminating contaminated dust from the renovated offices to the factory floor. The office block ran along the length of one end of the factory floor. Double-swing doors led from the second floor renovated offices onto an overhanging internal balcony 30 feet above the large open-plan factory floor (Figure 3). The factory production area had no windows and no air-conditioning system. A dust extraction system existed around some of the machines on the factory floor to collect waste cardboard. The lack of windows in the factory production area and the dust extraction system almost certainly caused a degree of negative pressure in the factory. This condition would draw air in through the double doors leading from the renovated office area and onto the factory floor.

## Discussion

Environmental and epidemiologic evidence suggests that this outbreak was associated with the renovation of an office block within a cardboard manufacturing plant. One potential source identified was straw board in walls and ceilings disturbed by the renovation work. If straw board had been contaminated at some time in the past with a concentrated source of *C. burnetii*, drilling into this could have produced a cloud of dust containing large numbers of *C. burnetii* sporelike forms. Dust containing *C. burnetii* sporelike forms could have been sucked through the balcony doors from the renovated offices, fallen onto the workforce below, and inhaled by those infected. Workers could also have been infected when visiting the personnel or accounts offices situated adjacent to the renovation work.

No record of visits to these departments exists, which would allow this hypothesis to be further assessed. However, the hypothesis is supported by a number of factors. The pattern of relative risk for infection in groups of participants at different machines on the factory floor is consistent with this hypothesis. The highest relative risks are in the center of the factory close to the balcony, while the lowest risks are in the areas at the sides and far end of the factory floor. The overhanging balcony may have sheltered employees at some of the machines from any contaminated dust falling from above. Raised ORs for infection in employees who were decanted into neighboring offices because their offices were being renovated, and in office staff whose offices had been refurbished, also implicate the renovation work as the source of the outbreak.

The timing of the installation of the new suspended ceiling (July 17–August 9, 2002) is consistent with an outbreak source near August 5 through August 9. The raised OR in persons rarely near an open window or door compared with those often near an open window or door and the lack of cases among those who worked in the separate design office, or among sales representatives, suggest that the source of the outbreak was inside the factory.

The respirable dust fraction that is most pathogenic is generally invisible to the naked eye (15,16). We do not have a good proxy for exposure in this outbreak, and consequently the issue of a dose response has not been addressed. Exact place of work probably did not closely correlate with exposure as many staff members move around the building as part of their work.

### Potential Contamination of the Straw Board

Straw board could have been contaminated either before or after manufacture. Investigating the process used to make the straw board indicated that the low pressures and temperatures involved would not kill any fungal spores present in the straw. If straw board becomes wet, these fungal spores often sprout and damage the board. The straw used to produce the board was stored in large Dutch barns and would have been accessible to rodents, cats, and other animals. Some evidence exists that a number of cases of Q fever were occurring around 1950 in the English county where the straw board was manufactured (17) and that the straw board was probably manufactured from 1950 to 1953. *C. burnetii* sporelike forms are resilient. They can withstand pressures of up to 20,000 lb/in^2^, elevated temperatures, desiccation, osmotic shock, UV light, and chemical disinfectants (18). However, experimental studies of the survival of *C. burnetii* spore-like forms have not demonstrated survival beyond 8 years (Table 6) (5,6). Whether experiments for longer durations were undertaken is not clear from the source documents. Although not directly comparable, *Bacillus anthracis* and *Clostridium tetani* spores are known to survive for many years. For example, *B. anthracis* spores have been recorded as surviving for 71 years on dried silk threads (19).

**Table 6:** Survival of *Coxiella burnetii*^a^

Environment	Temperature (°C)	Survival
Wool	15–20	7–9 mo
Wool	4–6	Approx. 12 mo
Sand	15–20	4 mo
Fresh meat	Cold storage	>1 mo
Salt meat	Not recorded	5 mo
Skimmed milk	Not recorded	40 mo
Tap water	Not recorded	30 mo
Tick feces	Room	Conclusive evidence: 586 d Some evidence: 6 and 8 y
Not recorded	–20	2 y
Not recorded	–65	8 y

^a^References 5 and 6.

Alternatively, the straw board could have been contaminated after manufacture by the feces, urine, birth products, or a corpse of an infected rodent that gained access to the inner layer of a straw board. Some holes were drilled in the straw board ceiling in 1982 and 1983, which could have provided a point of entry. Rodents are considered an important potential source of *C. burnetii*, and in one U.K. serosurvey, 34% of wild brown rats (*Rattus norvegicus*) had antibodies suggesting previous exposure to *C. burnetii* (20). The placentas of common rodents can also contain large numbers of *C. burnetii* sporelike forms (21) and could contaminate straw.

Test results of environmental samples in this outbreak were, however, negative. This finding could have occurred for a number of potential reasons. The samples were collected by persons who did not have detailed knowledge of the outbreak investigation, and the samples tested were minute in comparison to the quantity of straw disrupted during the renovation work. Concentration of potential bacterial contaminants was attempted in the PCR tests, but analysis was performed on small aliquots of extract, and bacterial DNA could therefore easily have been missed. The PCR test used was also experimental, although the protocol followed was similar to that used in Australia, France, and Germany. A delay of 2 months occurred between the dates when employees were probably exposed to *C. burnetii* and when environmental dust samples were collected. Consequently, contaminated dust may have been dispersed or cleaned up in the interim. In previous outbreak investigations, test results of environmental air or straw samples for *C. burnetii* have also more often been negative (15,22–23) than positive (4,24). The environmental sampling was, therefore, like looking for a "needle in a haystack."

### Other Hypotheses

We considered a range of alternative hypotheses but did not find any evidence to support them. For example, wind speeds were recorded routinely by the harbor authority but were very low during the week of August 5 through August 9, 2002, which makes windborne spread from the nearest farmland, 1 1/2 to 3 miles away, unlikely. No other potential wild or domestic animal sources were identified. Animals or animal products had not been moved through the docks in recent years. A feral cat had given birth in an adjacent building 1–2 years previously. One of the kittens had been adopted by an employee. However, the employee's serologic testing for Q fever was negative. If the feral cat had been infected with Q fever, the employee would most likely have had evidence of past exposure to *C. burnetii*. In addition, the factory strongly emphasizes controlling vermin as some of their cardboard packaging is used as secondary packaging in the food industry. No cats or other animals had been identified in or around the building for several years preceding the outbreak.

Contaminated fomites can produce secondary aerosols of *C. burnetii* sporelike forms (4), and several outbreaks have demonstrated the possibility of spread on fomites such as clothing, straw, hay, contaminated shoes, and building materials (22,25–36). However, unless a mechanism exists to repeatedly reaerosolize the source, fomites are likely to pose a risk even when they are not heavily contaminated, and this view is supported by the general principles that govern the dispersion and settling out of dust particles or sporelike forms (17,37).

Neither straw nor building material is a common source of outbreaks of Q fever. However, straw has been suggested as a possible source in several outbreaks (15,27,38,39). Two case reports implicate straw: a physician who contracted Q fever after clearing out straw and rubble from his new moorland home (26) and a businessman who was cleaning out a barn that had been used for keeping livestock 10 years previously but had not been properly cleaned since (24). Moldy hay from this barn, cultured using cell growth medium, grew *C. burnetii*. The renovation of buildings has also been suggested as a source of Q fever in two previous outbreaks (26,39). The widespread dispersal of spores in a building has been demonstrated both by Q fever (disseminated through a large medical school building) (23) and by anthrax (dispersed through a post office with an area of 281,387 ft^2^ and a volume of approximately 7 million ft^3^) (40).

One other alternate hypothesis is that the source of the outbreak was outside the factory building. Five persons mentioned having seen a hay lorry in the docks. This hypothesis was pursued because straw from farm vehicles had been implicated as a potential cause in a previous local outbreak of Q fever (2). However, the route taken by the lorries was never closer than half a mile to the factory. The lorries passed much closer to several other factories and to residential areas where several thousand persons would have had much greater exposure than the workforce at the factory. Although two Q fever cases were identified in the neighboring factory, no evidence existed of a wider outbreak involving other premises in the docks or nearby residential areas. The hypothesis that hay lorries passing through the docks could have caused the outbreak was known to a number of employees before they completed the questionnaire, and this finding may therefore be a result of diagnostic suspicion bias (41).

### Control Measures

Risk assessment and risk management was undertaken by identifying groups of persons at different levels of risk and providing relevant advice, temporarily stopping work in the area of the building considered at greatest risk, and following identified patients with Q fever. The cardboard manufactured by the factory was produced at temperatures that made survival of *C. burnetii* sporelike forms impossible so customers were not considered to be at increased risk. Unlike the straw board, which was produced a very low temperatures, the cardboard is produced at temperatures that would make survival of *C. burnetii* sporelike forms impossible. In addition, the cardboard was only used for secondary packaging and was therefore not in direct contact with any food products.

### Implications of the Study

Inhaled organic particles are an important source of a number of occupational diseases (17,42), and risks from exposure to occupational dust have been addressed by the U.K. Health and Safety Executive (43,44). Q fever is also a recognized occupational disease in the United Kingdom (45) and governed by existing legislation (46), although it is not a notifiable disease (47).

Straw is an increasingly popular ecologically friendly material, and >350,000 houses have been built in the United Kingdom with this particular type for straw board as internal partitions. The product has also been exported around the world. However, this outbreak is the first where straw board was suggested as a possible source of Q fever. Further research is needed to fully investigate straw board in various venues as a potential vehicle in Q fever outbreaks. Contaminated straw board represents a potential source of Q fever and should be considered in future outbreak investigations.
